# A91 CANADIAN FEMALE FELLOW PERSPECTIVES ON TRAINING IN THE BOYS CLUB: ADVANCED THERAPEUTIC ENDOSCOPY

**DOI:** 10.1093/jcag/gwab049.090

**Published:** 2022-02-21

**Authors:** A Jain, M Barker, J J Telford

**Affiliations:** Gastroenterology, The University of British Columbia, Vancouver, BC, Canada

## Abstract

**Background:**

Unequal female representation in the field of advanced therapeutic endoscopy (ATE) has been recently highlighted in the United States. Previous attempts to determine the barriers of entry into the career have found reasons including lack of mentorship, patriarchy, inflexible hours/call and exposure to fluoroscopy. There is no current literature describing the landscape of exposure to ATE for trainees in Canada or determining differences in experience based on gender.

**Aims:**

We sought to determine the barriers to pursuing a career in advanced therapeutic endoscopy, specifically focussing on the perspectives of Canadian female gastroenterology fellows.

**Methods:**

A survey was developed and distributed to the gastroenterology fellows enrolled in Royal College accredited programs across Canada via an online survey platform.

**Results:**

Responses were received from gastroenterology fellows at 12 out of the 14 Canadian universities with Royal College accredited programs. The response rate was 46% (n=42, 16 female respondents, 26 male respondents). An equal proportion of male (42%, n=11) and female (38%, n=6) trainees indicated interest in a career in ATE. 38% (n=6) of female trainees felt that they had inadequate mentorship opportunities/role models within ATE, in comparison to 4% (n=1) of males (p=0.004). Furthermore, 19% (n=3) of females felt that this lack of mentorship/role models was a primary deterrent from pursuing ATE as a career, in comparison to 0% of males (p=0.02). There was equal self-perceived competency surrounding ATE knowledge between both genders.

**Conclusions:**

Female gastroenterology fellows in Canada lack mentorship and role models in ATE, which they also indicated as a primary deterrent from pursuing it as a career when compared to their male counterparts. Recognizing and addressing the lack of female leadership and visibility is necessary to improve parity and encourage women to train in the male-dominated field of ATE.

**Funding Agencies:**

None

